# Supplementary material for: Stability and change in fertility intentions in response to the COVID-19 pandemic in Kenya
Source: PLOS Glob Public Health. 2022 Mar 8;2(3):e0000147. doi: 10.1371/journal.pgph.0000147 (PMC10021581; doi:10.1371/journal.pgph.0000147)
Supplement: S1 Table — (DOCX) [file pgph.0000147.s001.docx]

**Supplemental Table S1. Sample characteristics at baseline and follow-up for weighting procedures**

|  | | | **Baseline Eligible Sample**  **(N=5,204)** | | **Follow-up (Unweighted)**  **(N=3,095)** | | **Follow-up (Weighted*)**  **(N=3,095)** | |
| --- | --- | --- | --- | --- | --- | --- | --- | --- |
|  | | | **N** | ***%*** | **N** | ***%*** | **N** | ***%*** |
| ***Sociodemographic*** | | |  |  |  |  |  |  |
|  | **Residence** | |  |  |  |  |  |  |
|  |  | Urban | 1,465 | *28.4* | 1,096 | *35.4* | 864 | *27.9* |
|  |  | Rural | 3,699 | *71.6* | 1,999 | *64.5* | 2,231 | *72.1* |
|  | **Age** | |  |  |  |  |  |  |
|  |  | 15-24 | 906 | *17.6* | 471 | *15.2* | 640 | *20.7* |
|  |  | 25-34 | 2,189 | *42.5* | 1,357 | *43.8* | 1,255 | *40.5* |
|  |  | 35-49 | 2,069 | *40.1* | 1,267 | *40.9* | 1,200 | *38.8* |
|  | **Parity** | |  |  |  |  |  |  |
|  |  | Nulliparous | 192 | *3.7* | 69 | *2.2* | 78 | *2.5* |
|  |  | 1-2 | 1,803 | *34.9* | 1,115 | *36.0* | 1,137 | *36.7* |
|  |  | 3-4 | 1,763 | *34.1* | 1,161 | *37.5* | 1,094 | *35.3* |
|  |  | 5 or more | 1,406 | *27.2* | 750 | *24.2* | 785 | *25.4* |
|  | **Education** | |  |  |  |  |  |  |
|  |  | Never | 322 | *6.2* | 85 | *2.8* | 174 | *5.6* |
|  |  | Primary | 2,684 | *52.0* | 1,587 | *51.3* | 1,649 | *53.3* |
|  |  | Secondary or higher | 2,158 | *41.8* | 1,423 | *45.9* | 1,272 | *41.1* |
|  | **Wealth** | |  |  |  |  |  |  |
|  |  | Low | 1,944 | *37.6* | 885 | *28.6* | 1,172 | *37.9* |
|  |  | Middle | 1,727 | *33.4* | 1,194 | *38.6* | 1,044 | *33.7* |
|  |  | High | 1,493 | *28.9* | 1,016 | *32.8* | 879 | *28.4* |

*Note: *Weighting adjusted for age, residence, education, and wealth; and probability of phone ownership.*
