# Supplementary material for: Stability and change in fertility intentions in response to the COVID-19 pandemic in Kenya
Source: PLOS Glob Public Health. 2022 Mar 8;2(3):e0000147. doi: 10.1371/journal.pgph.0000147 (PMC10021581; doi:10.1371/journal.pgph.0000147)
Supplement: S2 Table — (DOCX) [file pgph.0000147.s002.docx]

**Supplemental Table S2. Bivariate associations between women’s characteristics and changes in fertility intentions**

|  |  |  | **Pronatal^**  **(n=1,624)** | | | **Antinatal†**  **(n=1,471)** | | | **Accelerate***  **(n=1,039)** | | | **Delay‡**  **(n=224)** | | |
| --- | --- | --- | --- | --- | --- | --- | --- | --- | --- | --- | --- | --- | --- | --- |
|  |  |  | **No** | **Yes** | **p-val** | **No** | **Yes** | **p-val** | **No** | **Yes** | **p-val** | **No** | **Yes** | **p-val** |
| ***Sociodemographic*** | | |  |  |  |  |  |  |  |  |  |  |  |  |
|  | **Residence** | |  |  |  |  |  |  |  |  |  |  |  |  |
|  |  | Urban | 79.6 | 20.4 | 0.463 | 86.4 | 13.6 | 0.567 | 90.8 | 9.2 | 0.739 | 53.0 | 47.0 | 0.968 |
|  |  | Rural | 81.1 | 18.9 |  | 87.6 | 12.4 |  | 89.8 | 10.2 |  | 52.7 | 47.3 |  |
|  | **Education** | |  |  |  |  |  |  |  |  |  |  |  |  |
|  |  | Primary or lower | 82.7 | 17.3 | 0.941 | 76.6 | 23.4 | **0.018** | 74.2 | 25.8 | **0.089** | 65.1 | 34.9 | 0.434 |
|  |  | Secondary or higher | 80.9 | 19.1 |  | 86.1 | 13.9 |  | 90.1 | 9.9 |  | 56.8 | 43.2 |  |
|  | **Parity** | |  |  |  |  |  |  |  |  |  |  |  |  |
|  |  | 1-2 | 55.3 | 44.7 | **<0.001** | 92.2 | 7.8 | **<0.001** | 90.4 | 9.6 | 0.527 | 53.9 | 46.1 | 0.708 |
|  |  | 3-4 | 77.2 | 22.8 |  | 80.2 | 19.8 |  | 90.8 | 9.2 |  | 45.3 | 54.7 |  |
|  |  | 5+ | 90.7 | 9.3 |  | 65.1 | 34.9 |  | 85.5 | 14.6 |  | 60.2 | 39.8 |  |
|  | **Wealth** | |  |  |  |  |  |  |  |  |  |  |  |  |
|  |  | Low | 81.2 | 18.8 | 0.856 | 87.2 | 12.8 | 0.563 | 89.0 | 11.0 | 0.752 | 51.9 | 48.1 | 0.271 |
|  |  | Middle | 81.3 | 18.7 |  | 86.2 | 13.8 |  | 91.1 | 8.9 |  | 45.0 | 55.0 |  |
|  |  | High | 79.9 | 20.1 |  | 88.9 | 11.1 |  | 90.4 | 9.6 |  | 59.5 | 40.5 |  |
| ***COVID-19-related factors*** | | | |  |  |  |  |  |  |  |  |  |  |  |
|  | **Household income loss** | | |  |  |  |  |  |  |  |  |  |  |  |
|  |  | None/Partial | 80.0 | 20.0 | 0.405 | 89.1 | 10.9 | 0.049 | 89.8 | 10.2 | 0.808 | 56.0 | 44.0 | 0.267 |
|  |  | Complete | 82.1 | 17.9 |  | 85.0 | 15.0 |  | 90.5 | 9.6 |  | 48.2 | 51.8 |  |
|  | **Food insecurity since COVID** | | |  |  |  |  |  |  |  |  |  |  |  |
|  |  | No | 80.9 | 19.1 | 0.261 | 88.3 | 11.7 | 0.340 | 90.4 | 9.6 | **0.011** | 52.0 | 48.0 | 0.193 |
|  |  | Chronic stable | 74.2 | 25.8 |  | 88.5 | 11.5 |  | 77.7 | 22.3 |  | 73.2 | 26.9 |  |
|  |  | Increased | 83.4 | 16.6 |  | 84.3 | 15.7 |  | 94.4 | 5.6 |  | 48.1 | 51.9 |  |
|  | **Able to socially distance** | | |  |  |  |  |  |  |  |  |  |  |  |
|  |  | No | 83.2 | 16.9 | 0.176 | 89.9 | 10.1 | 0.131 | 89.9 | 10.1 | 0.991 | 59.2 | 40.8 | 0.158 |
|  |  | Yes | 79.8 | 20.2 |  | 86.3 | 13.7 |  | 90.2 | 9.8 |  | 49.5 | 50.5 |  |
| *Notes: ^Shifts to pronatal fertility intentions explored only among women who reported wanting no more/no children at baseline. †Shifts to antinatal fertility intentions explored only among women who reported wanting any/more children at baseline. *Accelerating fertility intentions explored only among women who reported wanting children in more than one year at baseline. ‡Delaying fertility intentions explored only among women who reported wanting children within one year at baseline.* | | | | | | | | | | | | | | |
