# Supplementary material for: Stability and change in fertility intentions in response to the COVID-19 pandemic in Kenya
Source: PLOS Glob Public Health. 2022 Mar 8;2(3):e0000147. doi: 10.1371/journal.pgph.0000147 (PMC10021581; doi:10.1371/journal.pgph.0000147)
Supplement: Attachment — Submitted filename: Response to Reviewers.docx [file pgph.0000147.s003.docx]

We thank the reviewers and editor for their thoughtful and detailed feedback. We have provided a point-by-point response to each reviewer below. We hope that our responses are sufficient but are happy to provide any further clarification or revisions as necessary.

**JOURNAL REQUIREMENTS**

**1. During our internal checks, the in-house editorial staff noted that you conducted research or obtained samples in another country. Please check the relevant national regulations and laws applying to foreign researchers and state whether you obtained the required permits and approvals. Please address this in your ethics statement in both the manuscript and submission information. In addition, please ensure that you have suitably acknowledged the contributions of any local collaborators involved in this work in your authorship list and/or Acknowledgements. Authorship criteria is based on the International Committee of Medical Journal Editors (ICMJE) Uniform Requirements for Manuscripts Submitted to Biomedical Journals.**

*Response****:*** *Thank you for this comment. We note that Peter Gichangi and Mary Thiongo, the PIs of PMA Kenya, are both Kenyan nationals who were identified in the submission as being responsible for oversight of the project in Kenya. We clarified this further in the author contributions section. None of the other co-authors listed were directly responsible for conducting research or obtaining data within Kenya and, thus, no specific permits or approvals were required. Additionally, the primary IRB on record was the Kenyatta National Hospital-University of Nairobi Ethics Research Committee. Acknowledgement of the PMA Kenya team is already included in the acknowledgements section, however if additional acknowledgement is required, we are happy to expand. As data collection was overseen by Kenyan nationals and not by foreign researchers, we have not modified the ethics statement. If the editors require that we modify the statement, we are happy to do so, but appreciate guidance on what is needed as no foreign researchers were directly involved in data collection.*

**2. We note that participants provided oral consent. Please state in the Methods:**

*Response: We have expanded upon this in the manuscript in lines 274-301.*

- **Why written consent could not be obtained**

*Response: Data for our study were collected at the height of COVID-19 restrictions in Kenya. Face to face interviews were not allowed as a measure to prevent further spread of COVID-19, thus phone interviews were the only approved means of data collection. Only oral consent could be gathered via the phone.*

- **Whether the Institutional Review Board (IRB) approved use of oral consent**

*Response: We received a waiver due to the reasons outlined above.*

- **How oral consent was documented**

*Response: The consent was read out to the participants, and the participants were asked if they gave consent to participate. They were explained that unlike other surveys they had participate in, they were not going to get the consent form nor sign one. There was a check box in the ODK form on the smartphone for the enumerators to select if the participant agreed to participate.*

- **Whether consent was informed.**

*Response: Yes, the verbal consent was informed. Consent procedures were read out loud to the respondent before questions were asked. These included information about the survey being voluntary, potential benefits and risks, and that they could stop the survey or refuse to answer questions at any point.*

**3. You indicated that you had ethical approval for your study. In your Methods section, please ensure you have also stated whether you obtained consent from parents or guardians of the minors included in the study or whether the research ethics committee or IRB specifically waived the need for their consent.**

*Response: The research application to ERC was very specific and we requested a waiver for written consent and assent for all study participants. This was informed by COVID-19 restrictions which did not allow in-person data collection. Our survey was administered via phone.*

**4. Please include additional information regarding the survey or questionnaire used in the study and ensure that you have provided sufficient details that others could replicate the analyses. For instance, if you developed a questionnaire as part of this study and it is not under a copyright more restrictive than CC-BY, please include a copy, in both the original language and English, as Supporting Information.**

*Response: A copy of both questionnaires have been uploaded as Supporting Information. As we used publicly available data from PMA, we supplied the PMA questionnaires that were available on the website.*

**5. Please provide separate figure files in .tif or .eps format only, and remove any figures embedded in your manuscript file. If you are using LaTeX, you do not need to remove embedded figures.**

*For more information about figure files please see our guidelines:*

<https://journals.plos.org/globalpublichealth/s/figures>

*Response****:*** *Figure files have been uploaded separately.*

**6. Tables should not be uploaded as individual files. Please remove these files and include the tables in your manuscript file.**

*Response****:*** *Tables have been included at the end of the manuscript file.*

**7. We notice that your supplementary figures are uploaded with the file type 'Figure' and are therefore included in the PDF. Please amend the file type to 'Supporting Information' . Please ensure that all Supporting Information files are included correctly and that each one has a legend listed in the manuscript after the references list.**

*Response: The supplementary figures have been uploaded and included as “Supporting information” files with the resubmission.*

**8. We have noticed that you have uploaded supporting information but you have not included a list of legends. Please add a full list of legends for all supporting information files (including figures, table and data files) after the references list.**

*Response: Thank you for noting this missing information. We included a list of legends for all supporting files (i.e., figures, main tables, supplemental tables), which can be found on pg. 32 of the revised resubmission.*

**9. The link you provided in the Data Availability Statement returns no result . Please amend your Data Availability Statement and indicate where the data may be found.**

*Response:* Thank you for noting this, however we were able to access the website when we clicked on the link provided in the word document Data Availability Statement. We note that the datasets are available for download from the PMA website, once users create a profile. Multiple papers published within the PLOS journal system, including the papers listed below, were published with this same or similar data availability statement.

- *Zimmerman LA, Desta S, Karp C, Yihdego M, Seme A, Shiferaw S, et al. (2021) Effect of the COVID-19 pandemic on health facility delivery in Ethiopia; results from PMA Ethiopia’s longitudinal panel. PLOS Glob Public Health 1(10): e0000023.* [*https://doi.org/10.1371/journal.pgph.0000023*](https://doi.org/10.1371/journal.pgph.0000023)
- *Sarnak DO, Wood SN, Zimmerman LA, Karp C, Makumbi F, Kibira SPS, et al. (2021) The role of partner influence in contraceptive adoption, discontinuation, and switching in a nationally representative cohort of Ugandan women. PLoS ONE 16(1): e0238662.* [*https://doi.org/10.1371/journal.pone.0238662*](https://doi.org/10.1371/journal.pone.0238662)
- *Zimmerman LA, Shiferaw S, Seme A, Yi Y, Grove J, Mershon C-H, et al. (2019) Evaluating consistency of recall of maternal and newborn care complications and intervention coverage using PMA panel data in SNNPR, Ethiopia. PLoS ONE 14(5): e0216612.* [*https://doi.org/10.1371/journal.pone.0216612*](https://doi.org/10.1371/journal.pone.0216612)

**ADDITIONAL EDITOR COMMENTS (IF PROVIDED):**

**Dear Authors,**

**Thank you for submitting the manuscript in Plos Global Public Health. Before proceeding further, please address the reviewers’ comments as well as the following comments:**

**1. Please mention in which wave of COVID-19 pandemic in Kenya the research was conducted? Due to the rapidly changing nature of the pandemic this is very important to mention the wave of the pandemic.**

*Response****:*** *Data collection was done during the first wave of COVID-19, between May and July 2020. This is specified in the data section on ~ line 291.*

**2. Baseline data was collected by in person questionnaire and follow-up survey was done using telephone interview. As the authors employed two types of data collection technique, does it have any implications on the nature of information bias in the study?**

*Response: Thank you for this valuable question. In the limitations section, we added a sentence to indicate that two different modes of data collection were used (pg. 21, ~lines 645-649). Unfortunately, as all participants had both an initial face-to-face interview and a follow-up phone-based interview, it is not possible to isolate any potential biases this approach--the reliance on phone-based follow-up surveys--may have introduced to our study results. In our weighting approach, however, we were able to account for the likelihood that women owned phones, recognizing that these women are expected to differ from those who do not own phones. While this does not account for differential survey mode, with potential differences in reporting of fertility intentions based on face-to-face vs. phone-based surveys, it does account for potential selection bias of women included in the analytic sample.*

New text: “We note that using two different modes of data collection (phone versus face-to-face) may introduce differential responses based on mode of interview; however, as all respondents had face-to-face interviews first and phone-based interviews second, it is not possible to quantify any potential bias.”

**3. Please mention how the authors adjusted the survey weight and control for cluster effect during the analysis**.

*Response: We thank the editor for this comment. In the methods section (page 13, ~lines 430-439), we specify that we accounted for the complex survey design in analyses, including the clustering of women within enumeration areas (EAs) and applied survey weights .*

**4. Please mention how variables were selected in the multivariable binomial logistic regression?**

*Response: Variables were selected following the conceptual framework, as detailed in Figure 1 and on page 12,~ lines 390. We clarify that variables were not selected based on statistical significance (e.g., p<0.05 in bivariate analysis) and, instead, were chosen for inclusion in the multivariable binomial logistic regression models based on the authors’ theory-driven hypotheses about how the pandemic may have influenced women’s fertility intentions.*

**5. The study was conducted at the early part of pandemic and the findings do not reflect their intention as the pandemic progresses. This should be mentioned in the limitation.**

*Response****:*** *Thank you for the comment. We agree that this is a limitation of the data, which were collected during the initial months of the pandemic (May-June 2020), and included a description of this in the limitations section on page 21, ~ lines 640-646.*

**REVIEWER FEEDBACK**

**REVIEWER #1**

**I would like to congratulate the authors for the submission—I find the article clear, to the point, and insightful as to what is happening in a country of Sub-Saharan Africa in regards to fertility intentions during the COVID-19 pandemic. This study fills a critical knowledge gap, as the available research on this issue mainly focuses on high-income countries. The findings of this study would be considered useful not only for Kenya but also for other developing countries.**

**The statistical methods used in the manuscript are appropriate (including the calculation of the post-stratification weight). My major comments on the analysis, which can be addressed easily, are as follows:**

1. **The 1st COVID-19 case was confirmed on 12 March 2020, and the follow-up survey was conducted during May-July 2020. Can the authors elaborate on the timeline of COVID-related restrictions in Kenya (viz., cessation of movement in and out of major cities, school and/or business closures, lockdown/curfew, etc.) to clarify if the major restrictions were put into place before or after the follow-up survey.**

*Response****:*** *Thank you for the comment. We clarified in page 8, ~ lines 251-260 when COVID-19 related restrictions were instituted in Kenya and that follow-up data collection happened during the time of strictest restrictions.*

**In total, 36% of the baseline respondents were not included in the follow-up. The authors consider including a supplementary table to compare their basic characteristics to the analytic sample. If these two groups are similar, then the generalizability of the findings would be strengthened.**

*Response: Thank you for this comment about women lost to follow-up between the baseline and follow-up surveys. As requested by this reviewer, we added a supplemental table comparing the basic characteristics (unweighted and weighted) of the follow-up analytic sample, relative to the full, eligible baseline sample of women aged 15-49 who were married/in-union and not pregnant/infertile. Given differences in the two samples (see “Follow-up (Unweighted)” columns, Supplemental Table S1), the weighting procedures we used resulted in a final analytic sample that more accurately reflected the sociodemographic characteristics of the eligible baseline sample. As this reviewer suggests, the use of these survey weights in the analysis supported the generalizability of findings to better reflect the nationally representative baseline sample.*

*We have added this clarification at page 10, ~ line 325.*

*New text: There were no statistically significant differences in sociodemographic characteristics between the weighted baseline and follow-up surveys following the application of survey weights (Supplemental Table S1).*

2. **The authors tested for interactions between each combination of wealth, household income loss, and food insecurity. Tables 4 and 5 only listed Wealth x Food insecurity interaction term. Is this because the Wealth x HH income loss interaction term was not significant in the “test”?**

*Response****:*** *Thank you for the question. Yes, this was because the interaction term of Wealth and HH income loss was not significant. We have clarified this in the text on page 13, ~ line 426.*

3. **Tables 4 and 5 should include a marker for marginally significant (p<0.10) estimates since marginally significant results were highlighted in the text**

*Response****:*** *Thank you for the suggestion. As the reviewer suggested, we included a marker for marginally significant (p<0.01) in both tables. We included a note under Tables 4 and 5 to specify this designation for the adjusted odds ratios (aORs) presented in the tables, “P-values denoted *<0.05, **<0.01.”*

**Minor comments:**

- **For the ease of the readers, Table 1 can include predictors used in the analysis as well.**
- **Citation style and formatting require review.**
- **Tempo sub-section in results (pp.14-15, lines 359-366) needs to refer to Table 3.**
- **The manuscript needs a review for correcting typos (e.g., p.16, line 386).**

*Response: We thank the reviewer for these suggestions. For Table 1, we expanded on how the variables were operationalized in-text (~lines 360-393), so we did not make direct changes to the table itself, as we were concerned that this result in a very long table for the reader. The citation style and formatting will be updated per journal guidance. The tempo sub-section results have been updated to refer to the correct table (Table 3). A final review was conducted to ensure the typos identified were corrected. Thank you for these suggestions.*

**REVIEWER #2**

**This manuscript goes mostly in line with PLOS Global Health since this is an original research, not published elsewhere and attempted a robust analysis with available data. However, I believe there have been some misinterpretation of the results in the paper. The result and discussion section needs revision. But, more importantly, the timeline of the study does not seem to be sufficient to address the said objectives. Because the follow up time did not cover even any of the four Covid waves completely. The data were collected when the first wave just started. To me this is a big concern.**

*Response****:*** *We thank the reviewer for their comment. The intention of the original study, from which our data come, was to collect data on COVID-19 knowledge, attitudes, and practices to guide national response and, thus, the survey was launched within a few months of onset of the pandemic. Survey results were, indeed, used to inform national and subnational policies, as well as monitor trends in population needs during a time of significant disruptions to daily life, as the survey was one of few population-based studies available when very little was known about the status of reproductive health needs during the pandemic.*

*As such, we framed our results only as representative of changes occurring during the immediate onset of the pandemic (or early pandemic period) and noted that these findings are likely to change as the pandemic continued (page 21, ~lines 645). To provide further clarification to this point, we have revised the objectives to specify that we are only exploring changes associated with the onset of the pandemic (page 6, ~lines 202).*

*New text: Specifically, using data from a longitudinal study among women aged 15-49 in Kenya, this study aims to 1) describe the extent to which individual-level intentions for future children (quantum) and the time frame of intentions to have a child (tempo) change, and for whom, in the context of the onset of the COVID-19 pandemic, and 2) examine the effect that changes in economic and health security associated with the onset of the COVID-19 pandemic had on quantum and tempo of fertility intentions.*

*While our data are unable to address long-term responses to the pandemic, we believe that they are still valuable and offer a critical contribution to the public health field, as they provide important insight into how a global pandemic affected fertility behaviors in a time of extreme uncertainty. Publications in other premier journals, including Lancet Global Health (Wood et al “Need for and use of contraception by women before and during COVID-19 in four sub-Saharan African geographies: results from population-based national or regional cohort surveys) and BMJ Sexual and Reproductive Health (Karp et al “Contraceptive dynamics during COVID-19 in sub-Saharan Africa: longitudinal evidence from Burkina Faso and Kenya”) used this same dataset to explore the immediate impact of COVID-19 on family planning behaviors, underscoring the importance of understanding the immediate impact of the pandemic on health and health behavior.*

**REVIEWER #3**

**The manuscript is timely and relevant to a contemporary problem; Also, it is well written, logical, and easy to understand; and the study is well designed and employs acceptable methods. There are a few small details that require fixing.**

1. **In the manuscript, there is no definition of household wealth or ability to socially distance oneself.**

*Response: Thank you. We have included additional clarification of these measures (page 11, ~line 370-390).*

*New text (line 385): Household wealth, which is created and provided in PMA public use datasets and was not created independently for this analysis, was measured through the creation of a continuous score from an index of household assets, using a similar methodology as employed by the Demographic and Health Survey^40^. Tertiles were created based on the household score.*

*New text (line 369): Ability to social distance was defined based on self-report to the question, “Are you able to avoid contact with people outside your household?“ (Yes/No).*

2. **In order for other researchers to repeat the experiment, household food insecurity must also be described.**

*Response: Thank you. We have expanded on the definition of food insecurity in the description of the measures (see below)..*

*New text (~line 375): Food insecurity was defined relative to pre-COVID-19 using two survey items. First, we asked if the respondent or a household member went a whole day and night without eating since the pandemic began (yes/no), and, if so, whether this experience was more common than before COVID-19 (yes/no). We created a three-level categorical variable indicating no food insecurity, chronic food insecurity (food insecurity before and after COVID-19 that did not worsen), and increased food insecurity.*

3. **Instead of Table 2, the authors might include a bivariate table that includes women's characteristics and baseline fertility intentions.**

*Response: Thank you for this comment. We included the bivariate associations between women’s characteristics and changes in fertility intentions as a Supplemental Table S2.*

**REVIEWER #4**

**This manuscript investigates the effects of the COVID-19 pandemic on fertility intentions. The study was conducted using data from the Performance Monitoring for Action (PMA), a longitudinal data collected to examine key reproductive indicators in sub-Saharan Africa and South-East Asia. The current prospective study used the PMA data collected in November, 2019 and follow-up in June 2020 and examined how economic and health security due to the pandemic affected the change in quantum and timing of fertility intentions of 3, 095 women in Kenya. Exploratory analysis was used to describe the overall changes in the sample whilst logistic regression models were used to assess sociodemographic and COVID-19 related changes. The study reported no COVID-19 related factors associated with the quantum of childbearing. However, women who reported chronic food security had 4.78 times higher odds of accelerating their desired timing to next birth compared to those who reported no food insecurity. The study concluded that the COVID-19 pandemic did not lead to widespread changes in fertility intentions in Kenya, though vulnerable women may have accelerated their childbearing intentions.**

**The study addresses an important issue on the impact of the CoVID-19 pandemic in relation to women’s intention to give birth to more and the timing of these births. The study utilized data from the Performance Monitoring for Action survey which was conducted both before and during the CoVID-19 outbreak in Kenya. The study has key strengths including the robust design of the data collection process and the prospective nature of the data collected. However, the study has notable weaknesses that are worth addressing. These include:**

**1. The study’s title is misleading. The title talks about longitudinal exploration, and this is repeated in the discussion. However, the investigators applied logistic regression in their analysis. For a longitudinal exploration one would expect to see profile analysis, Generalized Estimating Equations (GEE) or mixed models as these will help explore the longitudinal as well as any cross-sectional effects. The investigators should clarify the use of longitudinal exploration in the title and discussions.**

*Response: We thank the reviewer for their question. We revised the title to remove mention of longitudinal exploration, and it currently reads, “Stability and change in fertility intentions in response to the COVID-19 pandemic in Kenya”. We used two rounds of data, gathered among the same sample of women at two different time points, to evaluate change in a stated behavior over time. As the outcome is related to change in fertility intentions, the longitudinal nature of the data were captured by creating the outcome, as cross-classified between the baseline and follow-up rounds. Though the reviewer does provide an excellent example of an alternative method of longitudinal analysis (GEE), we note that the interpretation would not be the same. We have outlined below the rationale for this decision.*

*Using a GEE model would require a “long” dataset with fertility intentions as the outcome (y: fertility intention: wanting any/more children=1 vs.wanting no more=0) and an interaction with time with t=0 if baseline, t=1 if follow-up (to adjust for clustering at individual level). Instead, the approach we have taken in the current analysis and manuscript, uses the model:“y_it = bo + b1FertilityIntention_it + b2Time_it + b3_FertilityIntention*Time + b4 Covariates….” We note that this approach is analogous to a difference-in-difference analysis and b3 would capture any changes in fertility intention.*

*The key distinction between the two modeling approaches is that the current analysis examined “directions” in fertility intention changes that were designated quantum (stable/aninatlist) and tempo changes, but in the GEE analysis, the b3 result would reflect whether overall fertility intention increased (b3 positive), decreased (b3 negative), or no change (b3=0, statistically) at the population level during COVID-19. As our intention was to capture factors associated with individual, rather than population-level change, we believe our current model is preferred.*

**2. The use of ordinary logistic regression limits the ability to assess the longitudinal effects of the impact of COVID-19 on the fertility intentions. It may be important to examine these using GEE. The investigators need to justify why they did not use longitudinal methods.**

*Response: Please see our response to item #1 raised by Reviewer #4 above.*

**3. The loss to follow up of 37.9% is so high to ignore. The investigators should explore and report, just like they did with the 6% of the women who reported they did not know if and when they will have another child, how those who lost to follow up differed from those who were part of the final analysis.**

*Response: As requested by this reviewer and Reviewer #1, we added a supplemental table comparing the sociodemographic characteristics (unweighted and weighted) of the follow-up analytic sample, relative to the full, eligible baseline sample of women aged 15-49 who were married/in-union and not pregnant/infertile. Given differences in the two samples (see “Follow-up (Unweighted)” columns, Supplemental Table S1), the weighting procedures we used resulted in a final analytic sample that more accurately reflected the sociodemographic characteristics of the eligible baseline sample. As this reviewer suggests, the use of these survey weights in the analysis supported the generalizability of findings to better reflect the nationally representative baseline sample. We note on page 10, ~ line 325 that once survey weights were applied there no differences in sociodemographic characteristics between the baseline and follow-up sample.*

*New text: There were no statistically significant differences in sociodemographic characteristics between the weighted baseline and follow-up surveys following the application of survey weights (Supplemental Table S1).*

**4. The COVID-19 pandemic outbreak occurred mostly in some urban areas in Kenya. It will be important to account for the areas where the study participants are coming from in relation to how severe the pandemic was. For instance, what’s the distribution of the study participants between the areas worst hit by the pandemic and those that are not? From the Table 2, it will appear that most of the study participants (72.1%) were from the rural areas. It will seem to suggest that since most of the hard hit areas were the urban centers, most participants in the study may not have really been affected by the pandemic. There is the need to account for this variability.**

*Response****:*** *We thank the authors for this thoughtful comment. Unfortunately, regionally specific data on the impact of COVID-19 in the early stage of the pandemic is not readily available so we cannot compare areas based on COVID severity. We do agree that the impact of COVID-19 is likely to vary between urban and rural women, which is why we included residence as an adjustment variable. Given sample size considerations, we were unfortunately not able to stratify by residence. We note that the COVID-19 mitigation measures that were put in place in the early stages of the pandemic covered both urban and rural areas. These mitigation measures were particularly strict from mid-March through mid-July as noted in page 8, ~ line 255, which covered the study period. We agree with the reviewer that the infectious disease epidemiology of COVID-19 was likely to differ significantly by residence but the presence of strict mitigation measures, and their impact on household means, food security, and fertility behavior, was relevant across the country.*

*Updated text: Several measures were taken within weeks of the first confirmed COVID-19 case to slow the spread of the pandemic, including social distancing and restrictions on gatherings, imposition of a night curfew, limitations on public transportation, and closure of schools^37^. At the time of follow-up data collection, detailed further below, nearly 3,000 cases of COVID-19 had been detected in Kenya, and face coverings were required in all public spaces^37^ Restrictions were strictest between mid-March and mid-June 2020 and lessened over time, however, with a resurgence in transmission beginning in mid-January, 2021, some restrictions have been re-imposed^37,38^. As of April 27, 2021, there were 156,981 cases and 2,643 deaths^39^.*

**5. Table 2 needs some further explanation. The table presents weighted columns totals and proportions. One would expect that since both measures are weighted, they will tally. However, the proportions do not tally with the totals. Please address this and present a clearer table.**

*Response: Thank you for noting this discrepancy in Table 2. We revisited the tables and recognized that unweighted Ns were previously presented; the note under the table was incorrect. To make this table clearer, we updated the tables, so both Ns and %s presented are weighted (page 28).*

**REVIEWER #5**

1. **The topic of the paper is much interesting and timely given the global and regional context of the COVID 19 scenario. This paper would also have significant influence of reproductive health policies and programs.**

**The introduction section, in general, looks good. However, I do emphasize on tightening the section and focusing more on the points of interest rather than having a detailed literature review. The methodology, result and discussion sections are well presented and elaborated.**

*Response: Thank you for your comment. We have shortened the introduction section slightly and focused more on literature specific to sub-Saharan Africa.*

2. **Some specific comments are as follows: Line 93: Please be specific what was anticipated.**

*Response: Thank you for this comment. We note that the anticipated estimtaes of increased unmet need and unintended pregnancy are provided in the paragraph immediately above this sentence. To avoid repetition, we did not include the estimates again.*

3. **Line 423: “research in high-income settings has suggested,”, need reference for this claim**

*Response: Thank you. We have included references to support this claim (page 17, ~line 545).*

**REVIEWER #6**

**The study has several strengths and significant implications. However, there are many key issues that should be addressed.**

**1. The title is misleading. In this study, longitudinal data were used to define the stability and change in fertility intentions variables. Indeed, instead of showing the longitudinal trend (e.g., over time such as Nov 2019 – June 2020, July–Dec 2020, Jan–June 2021, etc.), the authors explored the factors associated with stability and change in fertility intentions.**

*Response****:*** *Thank you for this comment. We revised the title to remove mention of longitudinal exploration, and it currently reads, “Stability and change in fertility intentions in response to the COVID-19 pandemic in Kenya”. We used two rounds of data, gathered among the same sample of women at two different time points, to evaluate change in a stated behavior over time. As the outcome is related to change in fertility intentions, the longitudinal nature of the data were captured by creating the outcome, as cross-classified between the baseline and follow-up rounds. Though the reviewer does provide an excellent example of an alternative longitudinal analysis, as we only have data gathered at two time points, such an analysis is not possible.*

**2. The Introduction is unnecessarily long. The authors discussed so many things but did not explain the appropriate context in Kenya.**

*Response****:*** *We thank the reviewer for their comment. We shortened the literature section in an effort to focus primarily on sub-Saharan Africa. Unfortunately there is little research available on the impact of COVID-19 on fertility intentions in sub-Saharan Africa in general and none in Kenya specifically, which limits our ability to summarize contextually relevant findings. However, we also believe this underscores the importance of contributing to this under-researched question.*

**3. PMA used a multiple-stage cluster sampling approach. Please mention the exact number of stages and elaborate on how data collection was done in each stage.**

*Response: We thank the reviewer for their comment. We have expanded on the description of the multistage cluster design and process of data collection on page 8, lines 265-271.*

*Revised text: This study draws on longitudinal data from Performance Monitoring for Action (PMA), a panel study designed to examine key reproductive health indicators in sub-Saharan Africa and South-East Asia. PMA used a multiple stage cluster sampling approach, starting with first randomly selecting 11 counties using probability proportional to size, then randomly selecting 308 enumeration areas (EAs) using probability proportional to size, stratified by county and urban/rural residence. Within each EA, enumerators compiled a list of all households and then randomly selected 35 households per EA. A household questionnaire was administered in each household, including a roster that identified all women age 15-49 who slept in the household the night before and/or are regular members of the household. The baseline PMA panel survey was implemented in November 2019 (herein referred to as “baseline”), during which women responded to an in-person questionnaire and shared information about their sociodemographic characteristics, reproductive histories, fertility intentions, and contraceptive behaviors. Women provided written informed consent for initial interviews and provided phone numbers to participate in follow-up surveys. Parental consent and adolescent assent were obtained for women younger than age 18.*

**4. Please use separate heading to define the outcome(s) and covariates.**

*Reponse: We thank the reviewer for their suggestion. We added a separate heading to this portion of the methods section to define the outcome(s) and covariates (page 10, line 332 and page 11, line 362).*

**5. The methods used are technically incorrect. The authors did not appropriately use the survey features (e.g., sampling weights, strata, and clusters).**

*Response: We thank the reviewer for their comment. We clarify that all analyses accounted for complex survey design, including through the application of weights and accounting for clustering (page 14, line 438).*

*Revised text: All analyses accounted for the complex survey design, using post-stratification weights and accounting for multistage selection and clustering. Analyses were conducted using Stata 16.1^41^.*

**6. Marginally associated is an awkward term. Suggest removing this term. Please interpret the results using the point estimate and 95% CI instead of focusing on p-values. The authors focused too much on statistical significance, which relies on sample size. However, some estimates seem to be clinically relevant (e.g., the odds ratio of 0.25), but the authors did not pay careful attention to explain these estimates.**

*Response: We thank the reviewer for their comment but note that other reviewers did not highlight the use of “marginally associated” as problematic. We refer to a number of fertility and family planning papers published in Plos One that use similar terminology when referring to relationships that approach, but do not reach statistical significance, to assure the reviewer that this language is commonly used in fertility and family planning research published at Plos. Additionally, as requested by Reviewer #1, we highlight results that are marginally significant in Tables 4 and 5 to align with results highlighted in-text.*

1. *Challa S, Shakya HB, Carter N, Boyce SC, Brooks MI, Aliou S, et al. (2020) Associations of spousal communication with contraceptive method use among adolescent wives and their husbands in Niger. PLoS ONE 15(8): e0237512.* [*https://doi.org/10.1371/journal.pone.0237512*](https://doi.org/10.1371/journal.pone.0237512)
2. *Sarnak DO, Wood SN, Zimmerman LA, Karp C, Makumbi F, Kibira SPS, et al. (2021) The role of partner influence in contraceptive adoption, discontinuation, and switching in a nationally representative cohort of Ugandan women. PLoS ONE 16(1): e0238662.* [*https://doi.org/10.1371/journal.pone.0238662*](https://doi.org/10.1371/journal.pone.0238662)
3. *Seifu CN, Fahey PP, Hailemariam TG, Atlantis E (2020) Association of husbands' education status with unintended pregnancy in their wives in southern Ethiopia: A cross-sectional study. PLoS ONE 15(7): e0235675.* [*https://doi.org/10.1371/journal.pone.0235675*](https://doi.org/10.1371/journal.pone.0235675)

*Regarding using the point estimate and confidence intervals rather than the p-value alone, we appreciate the reviewer’s comment, however, as the reviewer also rightly points out below, the confidence intervals are in some cases difficult to interpret given their size and the challenges imposed by our limited sample size. To avoid overstating our findings, we were cautious about interpreting results that did not achieve statistical significance. We have, however, noted the specific estimate to which the reviewer refers, given its potential clinical relevance, and expanded upon it in the discussion (page 18, line 565).*

*New text: Though not statistically significant, a similar relationship was observed when examining shifts towards delayed childbearing intentions, wherein women in the poorest households who experienced chronic food insecurity appeared least likely to report delaying their intentions.*

**7. Odds ratios seem to be overestimated, and some 95% intervals are uninterpretable. I suggest reporting the risk ratio. Besides, please report the risk difference (at least in the appendix), which is more important for target interventions.**

*Response: We understand the concerns raised by the reviewer related to wide confidence intervals. However, in cohort studies where the outcome is greater than 10% , the OR will exaggerate the risk ratio* [*[1, 2]*](https://www.zotero.org/google-docs/?Z06ulT)*. Though each outcome (e.g. having pronatal or antinatal intentions and accelerating or decelerating intentions) is less than 10% of the entire sample, when restricted to the analytic sample for each model (e.g. changing to having pronatal intentions among women who did not want more children, changing to having antinatal intentions among women who did want more children etc), the outcome is greater than 10%. As such, the risk ratios are likely to be overestimated.*

*While we agree that reporting the risk difference might be a consideration for studies focused on targeted interventions, the objective of this analysis was not to inform specific interventions related to changing fertility intentions. Rather our intention is to explore how COVID-19, measured through economic and food insecurity, was associated with these changing intentions. We also note that papers that have explored stability and change in intentions have used similar analytic strategies and models, and have not reported risk differences* [*[3–5]*](https://www.zotero.org/google-docs/?YMdfAl)*.*

1. [*Davies HTO, Crombie IK, Tavakoli M. When can odds ratios mislead? BMJ. 1998;316:989–91.*](https://www.zotero.org/google-docs/?3blSQ9)
2. [*Viera AJ. Odds ratios and risk ratios: what’s the difference and why does it matter? South Med J. 2008;101:730–4.*](https://www.zotero.org/google-docs/?3blSQ9)
3. [*Sennott C, Yeatman S. Stability and change in fertility preferences among young women in Malawi. Int Perspect Sex Reprod Health. 2012;38:34–42.*](https://www.zotero.org/google-docs/?3blSQ9)
4. [*Trinitapoli J, Yeatman S. Uncertainty and Fertility in a Generalized AIDS Epidemic. Am Sociol Rev. 2011;76:935–54.*](https://www.zotero.org/google-docs/?3blSQ9)
5. [*Trinitapoli J, Yeatman S. The Flexibility of Fertility Preferences in a Context of Uncertainty. Population and Development Review. 2018;44:87–116.*](https://www.zotero.org/google-docs/?3blSQ9)

**8. Model diagnostic is missing, which is very relevant in the context of this study.**

*Response: Thank you for this comment. We conducted Hosmer-Lemeshow tests to assess the goodness of fit for each logistic regression model we implemented. Test results for all models indicate that no model violated test assumptions (p>0.05), indicating good model fit. This was true for the models including the interaction terms as well. We added language about assessing model fit in the methods section (pg 13, lines 436-440.*

*New text: We assessed each model’s goodness-of-fit via Hosmer-Lemeshow tests; none violated test assumptions (p>0.05), indicating good model fit.*

**9. The first sentence of the Discussion doesn’t make any sense. If the authors refer to Table 3 for this sentence, I should say the results are confounded. Besides, widespread economic loss and increased food insecurity were not measured in this study but supported from the literature.**

*Response****:*** *Thank you. As we found in our descriptive analyses, the majority of women did not change their fertility intentions, and we believe this justifies our claim that there was little change. We appreciate the authors suggestion to present the adjusted results, but after taking the reviewers suggestion to modify the objectives, our preference is to present the population level descriptive analyses as we have done. Other reviewers did not suggest that we present adjusted results for the descriptive findings, thus we hope that this is acceptable to the reviewer. We also note that economic loss and food insecurity were assessed in this study through the inclusion of variables that measured household income loss and food insecurity, as noted in line 369-382 and presented in Table 2. Over 90% of the sample reported either partial or complete household income loss and approximately 30% noted either consistent or worsened food insecurity.*

**10. Practically speaking, there could be many time-varying covariates that influence the stability and change in fertility intentions. Even some variables considered in this study (e.g., food insecurity) could be time-varying. The authors should expand the context in the literature and limitations parts discussing this time-varying covariate issue.**

*Response: Thank you for this comment. We agree with the author that there are likely to be many time varying covariates that affect fertility intentions, however we note that the time between baseline and follow-up was approximately six months. Though there would be some expected births, divorces, and deaths during that time, on the whole these are not likely to be a significant number of observations. While we expect that wealth status is likely to change as a result of the pandemic, we believe we have captured this effect by the inclusion of the variable measuring household income loss and food insecurity. Thus we expect that rather than large scale changes to the socio-demographic variables such as parity and marital status, changes to fertility intentions are likely due to the wide-spread disruptions related to COVID-19. Further discussion to this point is included on page 20, ~lines 612-629.*

**11. Overall, the study aims vs. the results authors showed were inconsistent.**

*Response: Based on the reviewers comments below we have updated the study aims to read “Specifically, using data from a longitudinal study among women aged 15-49 in Kenya, this study aims to 1) describe the extent to which individual-level intentions for future children (quantum) and the time frame of intentions to have a child (tempo) change, and for whom, in the context of the onset of the COVID-19 pandemic, and 2) examine the effect that changes in economic and health security associated with the onset of the COVID-19 pandemic had on quantum and tempo of fertility intentions.*” (page 6, ~line 200-206”

**11a. The authors aimed to “assess the extent …” but showed the confounded results in Table 3. That means the authors only showed the unadjusted incidence of changes to fertility intentions. Exploring the incidence of changes to fertility intentions could be one aim, but it should be clear to the readers before showing the results.**

*Response: Thank you for this comment. We have modified the first aim to read “describe the extent” so that it is clear that our first aim is to explore the incidence of changes, as the reviewer noted.*

**11b. The authors aimed to “examine how changes …” but actually explored the factors associated with stability and change in fertility intentions. The term “how” in the aim made it ambiguous, or at least as a reader, I didn’t understand the authors’ explanation.**

*Response: We have modified the study aims as noted above. We hope that our modified aims alleviate the reviewer’s confusion but will make additional revisions if necessary.*

**12. Some minor comments include: Please explain how the household wealth variable was constructed.**

*Response: Thank you for the note. We have expanded on this in the Methods (page 12, line 387-391) and copied below.*

*“Household wealth, which is created and provided in PMA public use datasets and was not created independently for this analysis, was measured through the creation of a continuous score from an index of household assets, using a similar methodology as employed by the Demographic and Health Survey^40^. Tertiles were created based on the household score.”*

**13. Results in Table 3 are unadjusted and thus confounded. Please provide the adjusted estimates (at least age and complex design-adjusted estimates).**

*Response: We clarify that the estimates presented in Table 3 do account for the complex survey design and account for both weights and clustering. Though the reviewer requestesd estimates adjusted for age, our preference is to retain the original descriptive statistics as our first aim, as described above, was to describe the overall change. We believe it is important to provide the crude estimates as these are generally more interpretable for a lay audience.*

**14. Please use “multivariable model” instead of “multivariate model”.**

*Response: Thank you for this suggestion. This has been updated on page 16, ~line 497.*

**15. Please use “x times odds” or “(x-1)*100% higher odds”, not “x times higher”.**

*Response: Thank you for this suggestion. We reviewed the results and ensured that all odds were reported as recommended by this reviewer (e.g., changed from “4.7 times higher odds” to “4.7 times odds”.*

**16. Tables 4 and 5 could be improved. The authors could show the interaction estimates for each category (e.g., within lowest, none vs. chronic stable and none vs. increased, and within none, lowest vs. middle, etc.). The Publish package in R could be helpful.**

*Response: Thank you for this comment about table presentation. Unfortunately, we are unclear on the specific request of the reviewer and as such, have not yet updated the tables. Currently, we show each combination of the categories of variables. Is the reviewer requesting that rather than show the interaction terms as categorical, we show the main effect and interaction term? As noted, we used Stata for this analysis. The results as currently shown are those generated from using code x#y, which generates categorical variables based on each combination of terms, versus the main effect and interaction term models which would be generated using code x##y.*
